# Magnetic Resonance Imaging for Diagnosis of Mandibular Involvement from Head and Neck Cancers: A Systematic Review and Meta-Analysis

**DOI:** 10.1371/journal.pone.0112267

**Published:** 2014-11-14

**Authors:** Chunjie Li, Wenbin Yang, Yi Men, Fanglong Wu, Jian Pan, Longjiang Li

**Affiliations:** Department of Oral and Maxillofacial Surgery, State Key Laboratory of Oral Diseases, West China Hospital of Stomatology, Sichuan University, Chengdu, China; Georgia Regents University, College of Dental Medicine, United States of America

## Abstract

**Background:**

Diagnosis of mandibular involvement caused by head and neck cancers is critical for treatment. We performed a meta-analysis to determine the diagnostic efficacy of MR for distinguishing mandibular involvement caused by head and neck cancers.

**Methods:**

Thirteen databases were searched electronically and hand-searching was also done. Two reviewers conducted study inclusion, data extractions, and quality assessment of the studies independently. Meta-disc 1.4 and STATA 11.0 were used to conduct the meta-analysis.

**Results:**

16 studies involving a total of 490 participants underwent MR examinations and were accounted for in this meta-analysis. Among the included studies, 2 had high risk of bias, while the rest had unclear risk of bias. Meta-regression showed that the slight clinical and methodological heterogeneities did not influence the outcome (P>0.05). Meta-analysis indicated that the MR for the diagnosis of mandibular involvement had a pooled sensitivity (SEN) of 78%, specificity (SPE) of 83%, positive likelihood ratio (+LR) of 3.80, negative likelihood ratio (-LR) of 0.28, diagnostic odds ratio (DOR) of 28.94, area under curve (AUC) of 0.9110, and Q* of 0.8432. Two studies detected the diagnostic efficacy of MR for the mandibular medullar invasion, and only one study reported the inferior alveolar canal invasion, which made it impossible to include it in our meta-analysis. In comparing to CT, MR had a higher SEN without statistical significance (P = 0.08), but a significantly lower SPE (P = 0.04). The synthesized diagnostic efficacy (AUC and Q*) on mandibular involvement was similar between the two modalities (P>0.05).

**Conclusions:**

Present clinical evidence showed that MR had an acceptable diagnostic value in detecting mandibular involvement caused by head and neck cancers. MR exceeded CT in diagnosing patients with mandibular invasion (higher sensitivity than CT) but was less efficacious to exclude patients without the mandibular invasion (lower specificity than CT).

## Introduction

The treatment of locally advanced head and neck tumors is site specific, depending on the exact origin site, depth of invasion, involvement of surrounding tissues, and regional and distal metastasis [1]. A primary therapeutic choice is synthetic serial treatment, with radical surgery emphasized [2]. With excising tumors, losing part of healthy organs in both the oral cavity and neck cannot be avoided. Such surgical procedures thus induce cosmetic and functional problems [3]. Resection of the mandible or maxilla attracts the most attention from patients and clinicians once the cancer invades the jaw [4]–[5]. This is not only because the treatment modalities might be altered by the presence of jaw invasion [6]–[7], but also because the quality of life with a compromised from a mandibular resection is suboptimal [8]. Whether the mandible is involved should be carefully considered before surgery.

Diagnosis of bone invasion not only helps with the jaw resection decision, but also helps with discovering hidden or uncertain malignancies [9]. Clinicians now accept that mandibular or maxillary bone invasion should be a routine pre-operative procedure. Considering the complexity of the regional anatomy, a precise detection of the cancer together with the possible involved mandible should be considered. The choice of tools to detect mandibular involvement, however, is not standardized [10]–[11]. Conventionally used radiological tools are CT and MR, which easily reveal oral cancers and surrounding invaded tissues. Exactness of invaded mandibles revealed with these methods is unknown. We have recently published a systematic review concerning the efficacy of CT in distinguishing mandibular involvement and concluded that CT could partly fulfill this task [12]. Thus, in this systematic review, we are aiming to detect the diagnostic efficacy of MR for distinguishing mandibular involvement and depth (including medullary and inferior alveolar canal invasion) caused by head and neck cancers.

## Methods

The study inclusion, data extraction, and risk of bias assessment were conducted by two reviewers in duplicate. Any discrepancies were solved by introducing a third reviewer, the arbiter.

### Inclusion criteria

Any studies that met the inclusion criteria were considered eligible for this systematic review. (1) Types of studies: diagnostic test accuracy studies designed as cohort studies; (2) participants: patients with oral cancers or head and neck cancers at jaw-adjacent anatomical sites. Cancers were proved by pre-operative biopsy and suspended to have mandibular involvement from a clinical sign that they were within 2 cm from the mandible; (3) index tests: MR; (4) reference standard: pathological diagnosis; and (5) targeting conditions: invasion of the tumor to the mandible or inferior alveolar canal of primary head and neck cancers; (5) outcomes: true positive (TP), false positive (FP), false negative (FN), and true negative (TN).

### Search strategy

Electronic database and printed publications were included to retrieve relevant literature.

Bibliographic databases searches included: the Cochrane Oral Health Group's Trials Register (to Issue 3, 2013), The Cochrane Central Register of Controlled Trials (CENTRAL, via The Cochrane Library, to Issue 10, 2013), MEDLINE (via OVID, 1948 to Oct 14, 2013), EMBASE (via OVID, 1980 to 1948 to Oct 14, 2013), Cumulative Index for Nursing and Allied Health Literature (CINAHL, via EBSCO, 1980 to 1948 to Oct 14, 2013), Latin American and Caribbean Health Sciences (LILACs, via BIREME 1980 to1948 to Oct 14, 2013), Chinese BioMedical Literature Databases (CBM, 1978 to 1948 to Oct 14, 2013), China National Knowledge Infrastructure (CNKI, 1994 to 1948 to Oct 14, 2013), VIP database (1989 to 1948 to Oct 14, 2013), and Wangfang database (1998 to 1948 to Oct 14, 2013). Grey literatures were also searched: Science Paper Online (to 1948 to Oct 14, 2013), System for Information on Grey Literature in Europe (OpenSIGLE, 1980 to 2005), and WHO International Clinical Trials Registry Platform (WHO ICTRP, to 1948 to Oct 14, 2013).

Following the guidance of the Cochrane Handbook for Diagnostic Accuracy Reviews, draft version 0.4, search strategies for the bibliographic databases were designed and combined the MeSh terms with free text words [13]. The MeSh terms used included: “head and neck neoplasm”, “neoplasm invasiveness”, “jaw”, “mandibular nerve”, “magnetic resonance imaging”, and “sensitivity and specificity”.

A hand-searching project conducted in 2011 covering 21 Chinese dental journals aiming to classify clinical trials published from 2000 to 2010 were conducted and a database were created. A search for the databases was completed to retrieve relevant studies and references of the included studies were further searched for any other eligible studies.

The search records (titles and abstracts) were first scanned by two reviewers. All recognized records were combined and the full texts of those studies were retrieved. Full texts were further evaluated by the two reviewers based on inclusion criteria.

### Quality assessment

The quality assessment included a risk of bias assessment and applicability judgement via QUADAS-2 [14]. The assessment tool comprised four domains: patient selection, index test, reference standard, and flow and timing. Each domain was assessed in terms of risk of bias. The first three domains were additionally assessed in terms of concerns regarding applicability. Signaling questions were included to help judge risk of bias. The reviewers first read the full QUADAS-2 tool and then tailored it by either adding or omitting signaling questions. Review-specific guidance on how to assess each signaling question was developed to judge the risk of bias.

The signaling questions that remained in QUADAS-2 for this review included:

1. Patient selection:

Was a consecutive or random sample of patients enrolled?

Was a case–control design avoided?

Did the study avoid inappropriate exclusions?

1. Index test:

Were the index test results interpreted without knowledge of the results of the reference standard?

1. Reference standard:

Was the reference standard likely to correctly classify the target condition?

Were the reference standard results interpreted without knowledge of the results of the index test?

1. Flow and timing:

Was there an appropriate interval between index tests and reference standard?

Did all patients receive a reference standard?

Were all patients included in the analysis?

### Data extraction

A data extraction form was prepared and pilot-tested on five of the studies included. This form was based on one used in a CT review [12]. The content of the form included: Re-evaluation of eligibility; basic information of the study (authors, title, publication time, and correspondence); characteristics of the participants (age, gender, inclusion criteria, types of tumor, location of tumor, types of surgery, number of included patients, and follow-up); study location (country, source of patients); index test and reference standard (details of MR and pathological diagnosis, diagnosis criteria, blinding, and consistency of the radiologists); study design (types and duration of the study); and outcomes (TP, FP, FN, and TN of MR for mandibular involvement/inferior alveolar canal involvement).

### Meta-analysis

Meta-disc 1.4 and STATA 11.0 were adopted to perform the meta-analysis [15]–[16]. Studies were pooled when there were no significant clinical and methodological heterogeneities. Slight heterogeneities were detected by meta-regression when the number of included studies exceeded ten. Considering current research progress, reporting bias was not assessed [17].

### Statistical heterogeneity

The I^2^ test was used to explore statistical heterogeneity. Based on the recommendation by Cochrane Oral Health Group, if the number of studies in one meta-analysis exceeded four, the meta-analysis would be conducted via the random-effect model. Otherwise, the fixed-effect model would be considered.

### Meta-regression

Log Diagnostic odds ratio (logDOR) was considered as the dependent variable of meta-regression. Meta-regression based on single covariate was conducted at first, if P<0.05, this covariate would be considered in the following multi-covariates meta-regression. Clinical and methodological heterogeneities with potential to affect results were proof for subgroup analysis.

### Meta-analysis

The test standard for meta-analysis was set at α = 0.05. Data on diagnostic performance of MR, such as sensitivity (SEN), specificity (SPE), positive likelihood ratio (+LR), negative likelihood ratio (-LR), diagnostic odds ratio (DOR), and 95% confidence intervals (CIs), were quantitatively pooled. The summary reviewer operator characteristic curve (SROC curve) was drawn, and area under the curve (AUC) and Q* (the point of SROC on which sensitivity was equal to specificity) were calculated to reflect synthesized diagnostic accuracy. Descriptive qualitative analysis was adopted for data that could not be combined.

### Comparison between MR and CT

A Z-test was used to detect diagnostic differences between SEN, SPE, AUC and Q* of MR and CT. The formula used was: Z = (VAL_1_-VAL_2_)/SQRT(SE_1_^2^+SE_2_^2^). The VAL was the means of SEN, SPE, AUC or Q* of MR or CT, and SE was the standard error of corresponding variables. P<0.05 reflected statistical significance.

## Results

### Results of the search and study inclusion

**Figure 1:**
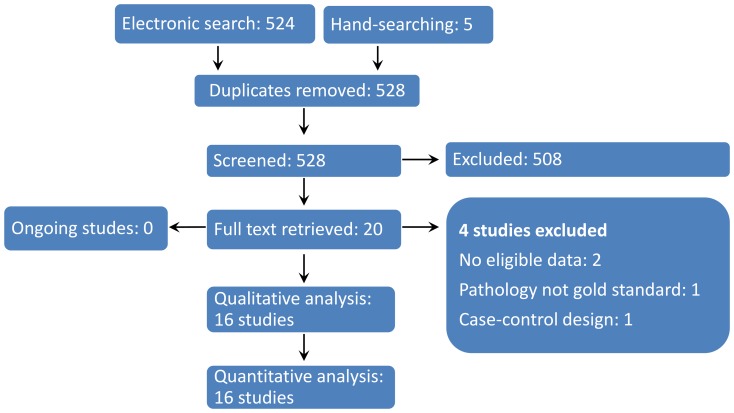
Flow diagram of study inclusion.

The number of search records retrieved was 528. After initial inclusion, 508 search records were removed and the remaining 20 articles were further evaluated by reading the full text. Finally, 16 studies were included [18]–[34] (Figure 1).

### Characteristics of the included studies

Among the 16 included studies, there were 6 prospective studies and 10 retrospective studies. 15 studies focused on the bone invasion (both cortical and medullary invasion) and 2 studies focused on medullary invasion (Chung 1994 [20] reported both bone invasion and medullary invasion results). A total of 598 patients were involved and 490 participants received an MR examination. For the 490 participants, bone invasion was confirmed for 249 of them. For detection of bone invasion by MR, there were 43 FPs and 51 FNs. For the use of MR, field strength ranged from 0.5 to 3T and the side thickness fell between 2.5–7 mm. All the included studies mentioned the ability of MR for the diagnosis of mandibular involvement, only one study explored the diagnostic efficacy of MR for inferior alveolar canal involvement. Details are presented in Table 1.

**Table 1:** Characteristics of included studies.

Study ID	Country	Study type	N (M/F)	Age Mean (range)	Tumor location	Number of patients got MR	No. of bone invasions
Bolzoni 2004 [18]	Italia	Prospective	43(37/6)	57(37–79)	Oral cavity, oropharynx	43	15
Brown 1994 [19]	UK	Prospective	35(28/7)	64.9	Oral cavity	14	11
Chung 1994 [20]	Netherland	Retrospective	22	-	Oral cavity, oropharynx	22	12
Gu 2010 [21]	Korea	Retrospective	46(39/7)	59.4(39–89)	Oral cavity	46	12
Hendrikx 2010 [22]	Netherland	Retrospective	23	63(43–84)	Oral cavity	23	11
Huang 2011 [23]	China	Prospective	17(16/1)	54(36–79)	Cheek	16	8
Imaizumi 2006 [24]	Janpan	Retrospective	51(39/12)	61(37–84)	Oral cavity	51	25
Kim 2013 [25]	Japan	Prospective	27(11/16)	73.6(53–90)	Oral cavity	27	20
Rajesh 2008 [26]	UK	Retrospective	23	-	Oral cavity	23	19
Smyth 1996 [27]	Ireland	Retrospective	40(33/7)	57(31–74)	FOM, RT, gingival, tonsil	8	3
Tsue 1994 [28]	USA	Retrospective	64(32/32)	62(26–78)	Gingiva, RT, FOM, cheek, tonsil, tongue, oropharynx	11	3
Van Cann 2008(A) [29]	Netherland	Prospective	67(42/25)	63(43–84)	FOM, RT, gingival, cheek	66	43
Van cann 2008(B) [30]	Netherland	Prospective	25(15/10)	54(48–76)	FOM, RT, gingiva	25	12
van den Brekel 1998 [31]	Netherland	Retrospective	29(19/10)	57(39–73)	Oral cavity	29	18
Vidiri 2010 [32]	Italia	Retrospective	36(26/10)	56(30–75)	FOM, gingiva, RT, lip	36	14
Zupi 1996 [33]	Italia	Retrospective	50(28/22)	-	Oral cavity	50	23

N: Number of included patients; M: Male; F: Female; FOM: Floor of mouth; RT: Retromolar trigone.

### Quality of the included studies

**Figure 2:**
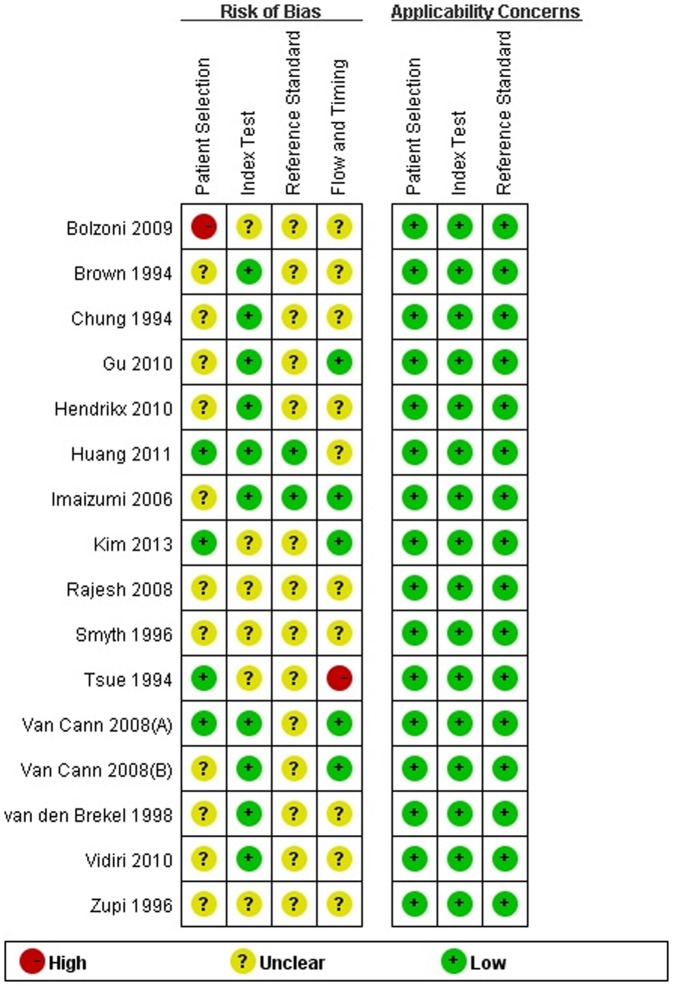
Risk of bias and applicability of included studies.

Two studies had high risk of bias and the rest had an unclear risk. Bolzoni 2004 [18] results were defined as high bias risk because of non-consecutive patient inclusion. Tsue 1994 [28] was considered as a high bias risk for problems in data presenting. All included studies had good applicability (Figure 2).

### Diagnostic efficacy of MR for mandibular involvement caused by oral cancers

#### Detection of mandibular involvement

Fiteen studies were considered. To investigate the potential heterogeneity, a meta-regression based on single covariate was conducted with the publication year (0 =  published before 2000; 1 =  published in or later than 2000), race (0 =  Mongoloid; 1 =  Caucasian), study type (0 =  retrospective; 1 =  prospective), percentage of mandibular involvement (0 =  lower than 50%; 1 =  more than 50%), field strength (0 =  lower than 1T; 1 =  higher than or equivalent to 1T), blinding of radiologists (0 =  no or unclear, 1 =  yes) and blinding of pathologists (0 =  no or unclear, 1 =  yes). Results indicated that these variables induced no significant heterogeneity (P>0.05) (Table 2). Based on this outcome, no multi-covariates meta-regression was conducted. Slide thickness was not included in meta-regression analysis, as nine studies did not report this data.

**Table 2:** Single covariate meta-regression results.

Variable	Coef.	Std. Err.	t	P	95% CI
Publication year	0.444	0.513	0.87	0.403	(−0.66;1.55)
Race	0.257	0.621	0.41	0.686	(−1.08;1.60)
Study type	0.448	0.563	0.79	0.441	(−0.77;1.66)
Percentage of bone invasion	−0.197	1.1290		0.8655	(0.06;11.09)
Blinding of radiologists	0.383	0.555	0.69	0.502	(−0.82;1.58)
Blinding of pathologists	−1.002	0.806	−1.24	0.236	(−2.74; 0.74)
Field strength	0.387	0.524	0.74	0.473	(−0.745;1.52)

**Figure 3:**
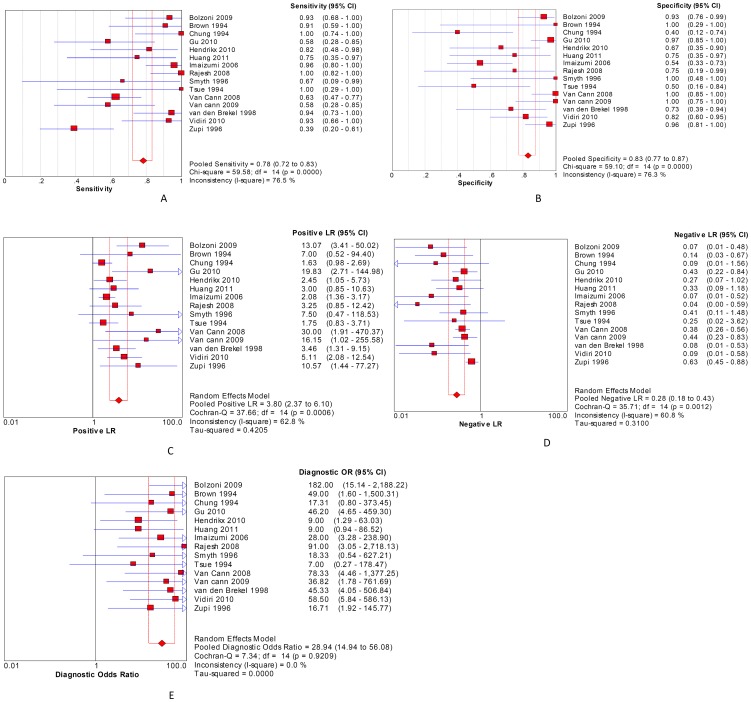
Results of the meta-analysis. A: SEN; B: SPE; C: +LR; D: -LR; E: DOR.

**Figure 4:**
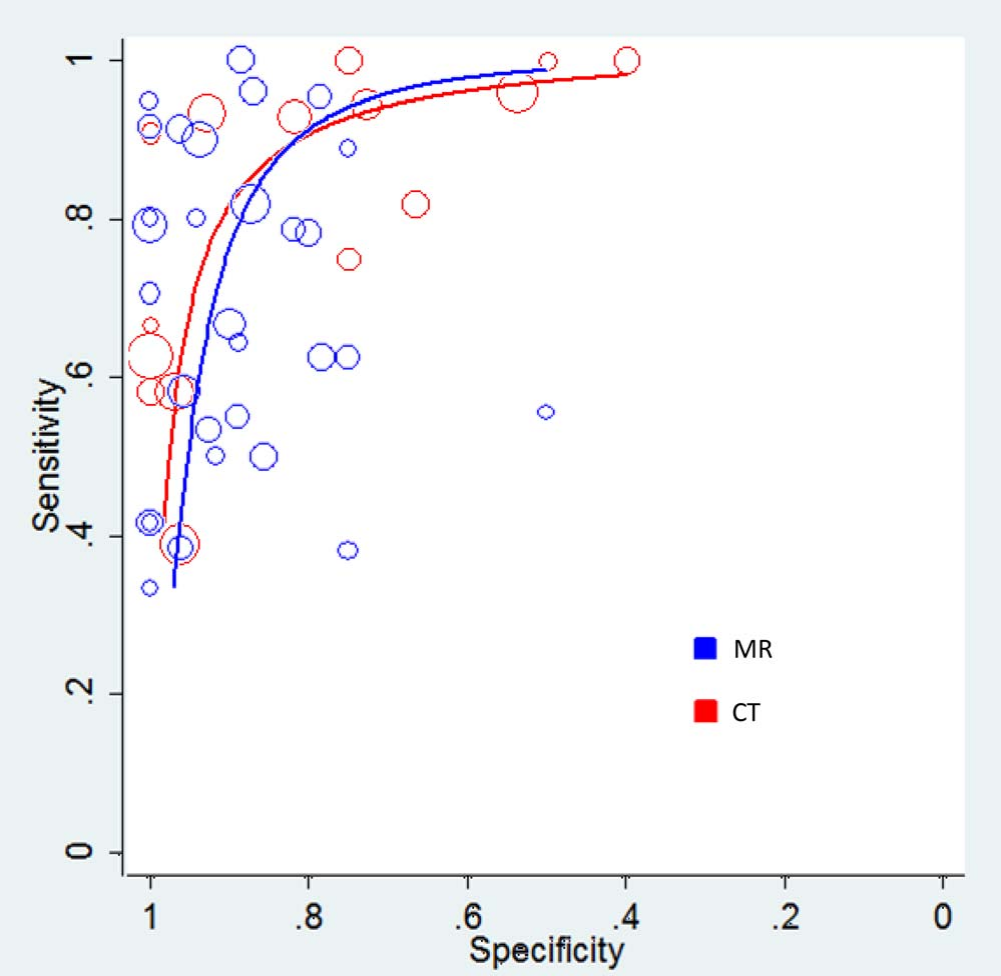
SROC curves of MR and CT for mandibular involvement diagnosis.

The meta-analysis showed that with a diagnosis of mandibular involvement by oral cancers, MR had a pooled SEN of 78% and 95% CI of (72%–83%), pooled SPE of 83% (77%–87%), pooled +LR of 3.80 (2.37–6.10), pooled –LR of 0.28 (0.18–0.43), DOR of 28.94 (14.94–56.08), AUC of 0.9110 and Q* of 0.8432 (Figure 3 and Figure 4).

Further investigations were made to see if the outcomes of the meta-analysis were stable. Sensitivity analysis was conducted by dividing included studies by slide thickness ≤3 mm, or >3 mm, excluding studies that did not report enhancement or had a high bias risk. Sensitivity analysis revealed an SEN between 0.807 and 0.851, an SPE between 0.755 and 0.821, AUC between 0.9029 and 0.9355, Q* between 0.8343 and 0.8718 (Table 3). Such outcomes predicted a grossly stable meta-analytic outcome.

**Table 3:** Sensitivity analysis.

	SEN (95% CI)	SPE (95% CI)	AUC (SE)	Q* (SE)
Slide thickness ≤3 mm	0.807(0.724–0.873)	0.755(0.660–0.835)	0.9029(0.0338)	0.8343 (0.0365)
Slide thickness >3 mm	0.851(0.758–0.918)	0.816(0.732–0.882)	0.9355(0.0248)	0.8718 (0.0306)
Exclude studies without reporting enhancement	0.807(0.741–0.861)	0.821(0.759–0.873)	0.9187(0.0211)	0.8519(0.0241)
Exclude high risk of bias studies	0.768(0.705–0.823)	0.828(0.768–0.878)	0.9061(0.0223)	0.8378(0.0244)

#### Detection of mandibular bone marrow invasion

Only two studies reported diagnostic efficacy of MR for bone marrow invasion, which prevented a meta-analysis. Data revealed that when a mandibular bone marrow invasion by oral cancers was diagnosed, MR showed an SEN around 95% to 100% and an SPE around 57.1% to 70.6% (Table 4).

**Table 4:** Diagnostic efficacy of MR for mandibular medullary involvement.

Study ID	TP	FP	FN	TN	Sensitivity	Specificity
Chung 1994	5	5	0	12	100%	70.6%
Kim 2013	19	3	1	4	95%	57.1%

#### Comparison between MR and CT

We compared diagnostic efficacy of bone invasion by MR with CT. Data for CT were derived from a systematic review recently published [12], which included 30 studies and involved 1459 participants. The comparison showed that MR had a slightly higher SEN than CT with no statistical significance (P = 0.08) and a significant lower SPE (P = 0.04). The summarized diagnostic efficacy, reflected by both AUC and Q*, showed that both CT and MR had approximately equal effects (P>0.05) (Figure 4). Detailed statistics are presented in Table 5.

**Table 5:** Comparison of results on diagnostic efficacy of MR and CT.

	SEN (95% CI)	SPE (95% CI)	AUC (SE)	Q* (SE)
MR	0.78 (0.72–0.83)	0.83 (0.77–0.87)	0.9110 (0.0201)	0.8432 (0.0223)
CT	0.72 (0.69–0.76)	0.90 (0.87–0.92)	0.9022 (0.0210)	0.8336 (0.0226)
Z	1.76	2.05	0.31	0.3
P	0.08	0.04	0.46	0.76

### Diagnostic efficacy of MR for inferior alveolar canal involvement caused by oral cancers

Imaizumi 2006 [24] reported the accuracy of MR for the detection of inferior alveolar canal involvement. Its SEN was 100% and SPE was 70%.

## Discussion

More than a million patients receive head and neck tumor diagnoses worldwide each year [34]. They are clinically characterized by diverse morphological features and pathologically characterized with marked local invasiveness [35]. All head and neck tumors share ability to invade the mandible. The prevalence of mandibular bone invasion by head and neck tumors ranges from 12 to 56% [28], [36]. Mandibular involvement influences the clinical staging of tumors, changes clinical treatment plans and alters prognosis [37]. Thus, diagnosis of mandibular involvement existence is believed to represent an important issue for pre-operative counseling and planning [32].

Normally, a pre-operative examination for head and neck tumors consists of clinical examination, imaging, and biopsy. Biopsy cannot elucidate mandibular involvement, so a pre-operative diagnosis of bone invasion relies on the other tests. Clinical examination can estimate mandibular involvement when the malignancy is within 2 cm of the mandible. Further confirmation via MR, CT, PET/CT [23], ortopanthomography, or CBCT is usually required [38]. Imaging reveals tumors, any metastasis, and mandibular involvement. Imaging also reveals invasion depth. If tumors invade mandible cortex, marginal madibulectomies, both rimly or sagittally, should be conducted [39]. This mandibulectomy resects the upper part of the mandible, including the alveolar process and teeth, but preserves continuity of the mandible [4]. But, continuity is not kept in a segmental mandibulectomy, for which a mandibular medullary involvement (deeper than cortex invasion) is diagnosed before surgery; and the involvement of inferior alveolar canal represents more extensive segmental mandibulectomy [40]. Adjusting mandibular continuity comes with a compromise on life quality [41]–[42]. So any misdiagnosis by the imaging techniques could induce a certain amount of mandible loss and seriously influence the life qualities. But any missed diagnosis could cause catastrophic outcomes. Therefore, accuracy of imaging techniques in diagnosis of mandibular involvement is critical.

MR is a frequently used imaging tool for diagnosing head and neck tumors [43]–[44]. It has great value in predicting mandibular involvement by head and neck tumors adjacent to or fixed to the mandible. MR easily detects invasion by highlighting signs of peripheral hypointense signal (cortical bone) replacement of the mandible through either tumor signal intensity on both T1- and T2-weighted images, or by replacement of central hyperintense signal (medullary bone) by intermediate tumor signal [21]. When the invaded medulla reached the inferior alveolar canal, it was considered as inferior alveolar canal involvement [24]. Included studies showed MR's SEN was between 39% and 100%, and SPE was between 40% and 100% when MR was used to diagnose mandibular involvement. Although such outcome diversities might derive from the different patient populations or different MR techniques, the variance led to a difficult judgment on the accuracy of MR on mandibular involvement diagnosis. So, to find a more precise answer how accurate that MR could detect the mandibular involvement, or to provide clinicians with solid evidence on behalf of MR to diagnose different types of mandibular involvement, a systematic review seemed critical.

A systematic review not only measures evidence credibility, but also assesses the putative factors that influence outcome. To fulfill the study aim, we thoroughly searched 12 bibliographic databases, 1 trial registration database, and 21 related dental journals. As mentioned above, 16 studies involving a total of 490 participants were included. These patients underwent MR examinations for mandibular involvement. We revealed that two studies involved a high bias risk. The risk for the remaining studies remained unclear. During the meta-analysis process, we first performed a meta-regression to judge if the factors related to the clinical or methodological heterogeneity could influence outcomes. We assessed the constitution of the participants' populations, design methods of the studies, MR parameters, and the quality of the studies. Results showed that none of these factors could influence outcomes. Such phenomenon revealed that some study outcomes may have been caused by chance instead of clinical or methodological heterogeneity. Or some factors may have been missed in the meta-regression or they could not be assessed. The former called for a meta-analysis based on its ability to pool data and eliminate as many chances as possible.

Three different meta-analyses were conducted quantifying mandibular involvement (both cortical and medullary invasion) and invasion depth (existence of medullary invasion). For the first part of the meta-analysis, 15 studies were included. The meta-analysis showed that MR had a pooled SEN of 78% and a pooled SPE of 83%. It also provided two variables that assessed the synthesized diagnostic efficacy, an AUC of 0.9110 and Q* of 0.8432. Both SEN and SPE reached a relatively high value, indicating that MR had high potency in the diagnosis of mandibular involvement. As mentioned previously, such outcomes may be altered by some clinical or methodological heterogeneities that we could not determine by meta-regression; thus sensitivity analysis was conducted by introducing those heterogeneities. Slide thickness can influence diagnostic accuracy. As some studies did not report this, it could not be addressed by meta-regression. We divided studies with slide thickness into two groups: ≤3 mm and >3 mm. It was clear that the sensitivity analysis result was similar to the meta-analysis, and the outcomes for slide thickness ≤3 mm was lower than for >3 mm. Thicker slides may provide more useful information, and well designed cohort studies should be conducted to compare different slide thicknesses. The other two meta-analysis focused on the diagnostic efficacy of MR in detecting depth of mandibular involvement (invasion into medulla or inferior alveolar canal). Only a few studies focused on these two issues resulting an SEN around 95% to 100% and an SPE around 57.1% to 70.6% in mandibular medullary invasion and an SEN of 100% and an SPE of 70% in inferior alveolar canal invasion. These results suggested that MR might have high SEN in invasion depth reorganization but the SPE was not satisfactory.

Typical preoperative imaging technique includes CT and MR [45]. Clinicians sometimes choose these two methods randomly. When the focus is on mandibular involvement, whether to choose CT or MR should be carefully considered. We have thus compared diagnostic efficacy between the two modalities. Synthesized diagnostic efficacy variables, AUC and Q* show that the two modalities share similar diagnostic efficacy. In comparing SEN and SPE separately, MR showed a relatively higher SEN than CT. Although the difference was not significant, but overlapping of their 95% CIs was quite slight. This may result from the imbalance in patient numbers: 490 in the MR group and 1459 in the CT group. MR exhibited better efficacy in distinguishing patients with mandibular involvement. CT showed a significant higher SPE value, indicating that CT is a better method to exclude a mandibular involvement diagnosis.

The second part of our meta-analysis involved investigating the medullary invasion. Since we found only two studies reporting such data, a meta-analysis was not conducted. MR had a high SEN and SPE was relatively low. No definite conclusions were made based on the limited trial numbers.

Although MR is an acceptable method for diagnosing mandibular involvement, the SEN and SPE could not reach an extremely high value. FPs and FNs existed. MR is prone to errors in cases of periapical and periodontal disease, and during remodeling after trauma or tooth extraction. This causes FPs in MR assessment. As for FNs, artifacts mask signs of bone invasion. Slide intervals of MR influence FN numbers. Larger slice intervals missed bone invasion and induced FNs, but FNs are rare with bone marrow invasion. This could have been due to the fact that for such patients, the area of bone invasion was extensive and could not be easily missed. This was considered to be the reason as to why the SEN of the medullary invasion diagnosis was higher than that of the cortical invasion. This hypothesis could not be assessed in this review, as most studies did not report slide intervals.

Bias existed during the review process. We may have unintentionally omitted studies, while others were impossible to retrieve. Bias risks were present in included studies, which influenced conclusion credibility. Clinical heterogeneities influenced outcome. Although we conducted a meta-regression to detect whether clinical variables influenced results, some variables could not be quantitatively detected. These included diagnostic ability of radiologists and special, unreported clinical variables. Inherent diversity amongst studies, small sample sizes, and high risk for biases were limitations. And the number of studies for assessing its ability in identifying mandibular medullary and inferior alveolar canal invasion was low which called for detailed investigating by the future studies.

## Conclusions

Present clinical evidence showed that MR has an acceptable diagnostic value in detecting mandibular involvement caused by head and neck cancers. MR exceeded CT in diagnosing patients with mandibular invasion (higher sensitivity than CT) but was less efficacious to exclude patients without the mandibular invasion (lower specificity than CT).

## Supporting Information

Checklist S1 **PRISMA checklist.**
(DOC)
